# Can the cyanide metabolite, 2-aminothiazoline-4-carboxylic acid, be used for forensic verification of cyanide poisoning?

**DOI:** 10.1007/s11419-024-00690-4

**Published:** 2024-05-13

**Authors:** Abdullah H. Alluhayb, Carter Severance, Tara Hendry-Hofer, Vikhyat S. Bebarta, Brian A. Logue

**Affiliations:** 1 Department of Chemistry, Biochemistry and Physics, Avera Health and Science Center, South Dakota State University, 1055 Campanile Ave, Box 2202, Brookings, SD 57007 USA; 2 Department of Chemistry, College of Science, Qassim University, Box 1162, Buraidah, 51452 Kingdom of Saudi Arabia; 3 Department of Emergency Medicine, University of Colorado School of Medicine, Aurora, CO 80045 USA

**Keywords:** Cyanide poisoning, Biomarkers of cyanide exposure, Thiocyanate, 2-amino-2-thiazoline-4-carboxylic acid, Forensic analysis

## Abstract

**Purpose:**

Forensic verification of cyanide (CN) poisoning by direct CN analysis in postmortem blood is challenging due to instability of CN in biological samples. CN metabolites, thiocyanate (SCN^−^) and 2-aminothiazoline-4-carboxylic acid (ATCA), have been proposed as more stable biomarkers, yet it is unclear if either is appropriate for this purpose. In this study, we evaluated the behavior of CN biomarkers in postmortem swine and postmortem blood to determine which serves as the best biomarker of CN exposure.

**Methods:**

CN, SCN^−^, and ATCA were measured in postmortem swine (*N* = 8) stored at 4 °C and postmortem blood stored at 25 °C (room temperature, RT) and 37 °C (typical human body temperature, HBT).

**Results:**

Following CN poisoning, the concentration of each CN biomarker increased well above the baseline. In postmortem swine, CN concentrations declined rapidly (*t*_1/2_ = 34.3 h) versus SCN^−^ (*t*_1/2_ = 359 h, 15 days) and ATCA (*t*_1/2_ = 544 h, 23 days). CN instability in postmortem blood increased at RT (*t*_1/2_ = 10.7 h) and HBT (*t*_1/2_ = 6.6 h). SCN^−^ and ATCA were more stable than CN at all storage conditions. In postmortem swine, the *t*_1/2_s of SCN^−^ and ATCA were 15 and 23 days, respectively. While both the t1/2s of SCN^−^ and ATCA were relatively lengthy, endogenous levels of SCN^−^ were much more variable than ATCA.

**Conclusion:**

While there are still questions to be answered, ATCA was the most adept forensic marker of CN poisoning (i.e., ATCA produced the longest half-life, the largest increase above baseline levels, and most stable background concentrations).

**Supplementary Information:**

The online version contains supplementary material available at 10.1007/s11419-024-00690-4.

## Introduction

Cyanide (HCN(g), HCN(aq), and CN^−^ all inclusively represented herein as CN) is a potent lethal chemical agent due to its toxic characteristic profile (i.e., CN inhibits cellular respiration and blocks the electron transport chain of cytochrome c oxidase) [1–6]. Because of its toxicity, ease of use, and ready availability (e.g., approximately 750,000 tons of CN is produced annually for extensive industrial applications in the U.S.) [7, 8], CN has been commonly used as a poison. In cases of CN poisoning, the determination of the cause of death has long been recognized as a significant and continuing problem in the field of forensic toxicology [2, 6, 9–24].

CN poisoning can occur through various routes of exposure, such as inhalation, ingestion, or skin contact [2, 7, 8, 23, 25–27]. The clinical symptoms of CN poisoning (e.g., chest pain, confusion, dizziness, eye pain, difficulty breathing, headache, abdominal cramping, nausea, rapid or slow heart rate, rapid or slow breathing, restlessness, shortness of breath, vomiting, weakness, and wheezing) are generally nonspecific, and often mimic other common illnesses such as flu or food poisoning [2, 4, 5, 7, 8, 23, 25, 26]. Therefore, CN poisoning can be easily confused with other medical conditions or causes of toxicity or death [19, 25, 28–30]. More specific indicators of CN poisoning are a “bitter almond” odor from the victim and pink lividity (i.e., a pinkish or rosy skin discoloration during postmortem examination). These indicators may help investigators identify CN-related fatalities but are difficult to identify, may rapidly disappear, and may be difficult to detect for certain individuals [2, 23, 26, 27, 31]. For example, pink lividity can arise from alternative conditions such as carbon monoxide poisoning. In addition, the olfactory smell of bitter almonds is contingent upon highly variable circumstances, including the quantity of CN present and its metabolic breakdown. It is also estimated that only 60% of people can recognize the “bitter almond” smell of CN [22, 30, 32–36]. Because of the nature of CN poisoning, it is often not immediately apparent to investigators that CN may have been utilized. Consequently, the collection and/or analysis of blood samples, which can serve as crucial evidence, is typically delayed until there is a growing suspicion of cyanide involvement [37, 38]. The duration of this delay can vary significantly, ranging from a few days to several weeks before samples are finally secured for analysis [31, 38, 39]. In addition, even if collection of blood is done immediately, it may not be analyzed for days, and in the interim, may be stored under non-ideal conditions.

While it is possible to directly analyze CN from biological samples in an attempt to verify CN poisoning, it is difficult to use CN as a biomarker in all but the earliest times following exposure [2, 27, 40]. This is because CN is highly unstable in biological matrices due to its chemical characteristics, including reactivity, volatility, and active metabolism of CN by normal biological processes. CN readily participates in certain reactions due to its nucleophilic nature. CN can bind tightly to iron and other metals and reacts readily with disulfides and thiol moieties in biological molecules [2, 10, 27]. The volatility of CN is another factor contributing to its loss from biological samples. CN exists predominantly as HCN(aq) under biological conditions (pK_a_ = 9.3 under standard conditions, and 9.2 at 37 °C). If blood samples are not properly handled and stored, HCN(aq) can quickly off gas from the sample [2, 3, 10, 27, 41]. Aside from its chemical reactivity and volatility, CN is metabolized efficiently by the human body [i.e., the half-life (*t*_1/2_) of CN in humans ≈ 20–60 min]. This rapid metabolism and chemical reactivity often return CN to background levels quickly [2, 27, 42]. If there is either an extended postmortem period before collection of samples or inadequate/prolonged storage before analysis, CN concentrations will likely decrease considerably, potentially back to baseline [2, 27, 43]. Another problem inherent in CN determination is the presence of small (<10 µM), but ubiquitous, endogenous concentrations in biological samples. These CN concentrations are present based on diet and natural metabolic processes [2, 4]. Therefore, to link CN poisoning to toxicity or death, blood concentrations of CN must be significantly elevated above background levels. Overall, the chemical and biological characteristics of CN make its direct use as an indicator of CN poisoning challenging unless blood samples are gathered, properly stored, and analyzed soon after exposure, which is not possible in most cases [2, 38].

**Fig. 1:**
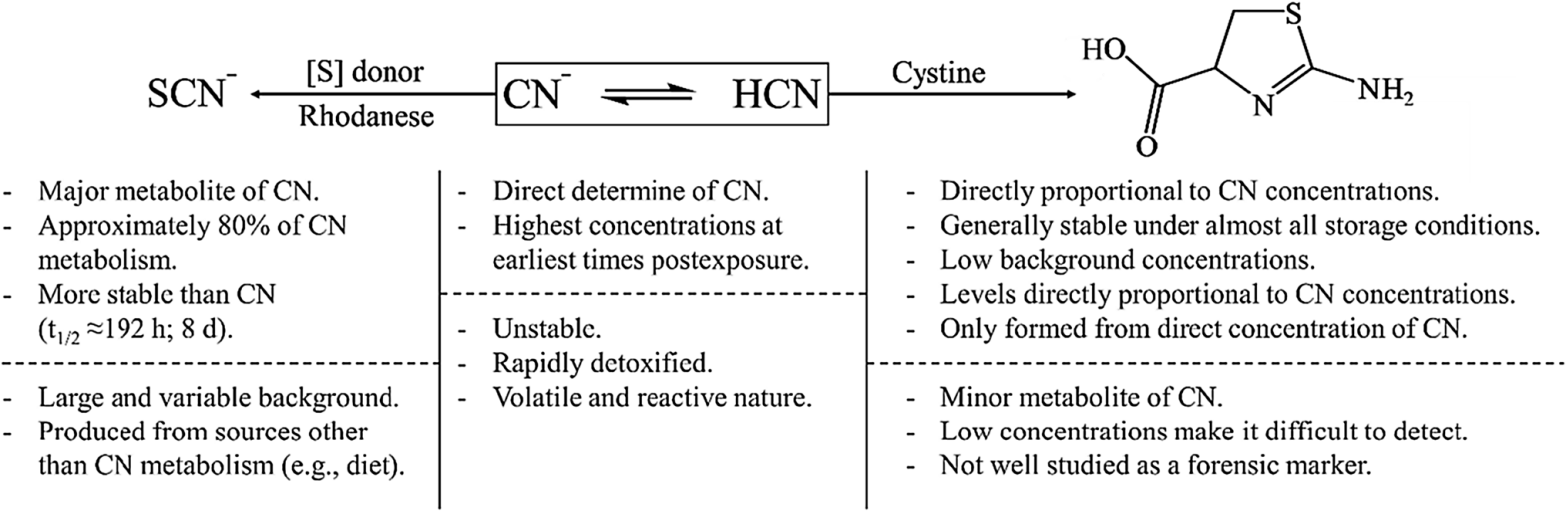
Characteristics of the biomarkers of CN exposure for forensic analysis. Dashed lines separate characteristics which are advantages (above the dashed line) and disadvantages for use of the biomarker for forensic purposes

An alternative to analyzing CN directly is to analyze its metabolites, thiocyanate (SCN^−^) and 2-aminothiazoline-4-carboxylic acid (ATCA), as biomarkers of CN poisoning (Fig. 1) [2, 39, 40]. SCN^−^, the major metabolic product of CN, accounts for approximately 80% of CN metabolism [44–46]. It is formed through a rhodanese-catalyzed reaction of CN with a sulfur donor, such as thiosulfate. Compared to CN, SCN^−^ is approximately 200 times less toxic and exhibits a longer half-life (*t*_1/2_ ≈ 96–192 h), making it a more persistent biomarker for CN exposure [2, 3, 5]. However, while SCN^−^ offers the advantages of abundance and stability, there are significant limitations for its use as a biomarker for CN exposure mainly due to its large and highly variable background concentrations [2–4, 27]. Notably, SCN^−^ is naturally present in common food sources and can arise from biological processes unrelated to CN metabolism [1, 2, 40]. Consequently, elevated levels of SCN^−^ may not result from CN poisoning and do not necessarily correlate with CN toxicity, especially under uncontrolled conditions [2, 4, 5, 32, 39, 47]. Therefore, distinguishing between elevated SCN^−^ concentrations due to CN exposure versus other natural sources is challenging. While SCN^−^ can be valuable in conjunction with blood CN concentrations and clinical symptoms, it has not been proven effective as a useful biomarker of CN exposure.

The in vivo conversion of cyanide to ATCA (Fig. 1), via direct reaction of CN with cystine, was initially reported by Wood and Cooley. [48] ATCA is a marker of CN exposure which is generally stable under almost all storage conditions and has low background concentrations in biological fluids [2, 32]. ATCA accounts for only a small amount of CN metabolism, but the concentration of ATCA is directly proportional to CN concentrations [2, 32, 39]. Moreover, besides the cyanide-mediated pathway, no other endogenous pathways for ATCA production within the human body have been identified [27, 32, 39, 49]. Extended studies of ATCA stability in biological samples have shown remarkable preservation of ATCA concentrations for prolonged periods at all temperatures tested [2, 27, 49]. Consequently, ATCA has garnered significant attention as a promising biomarker for forensic determination of CN poisoning.

While evidence is mounting that ATCA may be a highly stable and reliable marker of CN exposure, the stability of ATCA in biological samples has only been evaluated under controlled storage conditions, which may not be similar to postmortem decay. Therefore, the objective of this study was to determine if ATCA is valuable as a marker to verify CN poisoning under typical situations where blood samples are not immediately obtained and/or analysis is delayed. In addition, the behavior of CN and SCN^−^ was also evaluated. By examining these biomarkers in parallel, our study aimed to directly assess their interrelationships, offering valuable insights into their dynamics and their advantages and disadvantages as indicators of CN poisoning under the identical conditions.

## Experimental

### Materials

#### Reagents

All reagents used in this study were of analytical standard grade. The solvents, HPLC-grade methanol (MeOH), phosphoric acid (H_3_PO_4_, 85%), ammonium hydroxide (NH_4_OH, 29% by weight), LC–MS-grade and HPLC-grade acetonitrile (ACN), formic acid (CH_2_O_2_, 98%), and ammonium acetate (C_2_H_3_O_2_NH_4_) were purchased from Fisher Scientific (Fair Lawn, NJ, USA). Potassium cyanide (KCN), sodium hydroxide (NaOH), sulfuric acid (H_2_SO_4_), potassium dihydrogen phosphate (KH_2_PO_4_), and dibasic potassium phosphate (K_2_HPO_4_) were purchased from Fisher Scientific (Hanover Park, IL). Potassium thiocyanate (KSCN) was purchased from Acros Organics (Morris Plains, NJ, USA). 2,3-Naphthalene dialdehyde (NDA) was obtained from TCI America (Portland, OR). 2-Aminoethane sulfonic acid (taurine) and sodium metaborate tetrahydrate (NaBO_2_·4H_2_O) were purchased from Alfa Aesar (Ward Hill, MA). Monobromobimane (MBB) was purchased from Fluka Analytical through Sigma-Aldrich (St. Louis, MO, USA). Ellman's reagent (5,5′-dithiobis-2-nitrobenzoic acid) was obtained from Thermo Scientific (Hanover Park, IL, USA). Isotopically labeled internal standards, NaS^13^C^15^N and Na^13^C^15^N, were acquired from Isotech (Miamisburg, OH, USA). The ATCA internal standard (C_3_^13^CH_6_^15^N_2_O_2_S; ATCA-^13^C,^15^N_2_) was obtained from Toronto Research Chemical, Inc. (North York, Canada). The mixed-mode cation-exchange sorbent (Oasis-MCX®) was purchased from Waters Corporation (Milford, MA, United States). Ammonium formate (NH_4_HCO_2_) was purchased from Sigma-Aldrich (St. Louis, MO, USA). Pyrrolidine was purchased from Acros Organics (Morris Plains, NJ, USA). Purified water with a resistivity of 18.2 MΩ-cm was obtained using a water PRO PS polisher from Labconco (Kansas City, KS, USA).

#### Standard solutions

Phosphate borate buffer (0.01 M; pH 8.0) and NaOH (0.01 M) were prepared in deionized water and placed into a plastic bottle and stored on the benchtop. A solution of H_2_SO_4_ (2 M) was prepared using deionized water and ethanol and was kept at room temperature for storage. A stock solution of NDA (0.002 M) was prepared in a phosphate/borate buffer (0.01 M; pH 8.0) along with 40% methanol. NDA was dissolved completely in methanol before adding the buffer to ensure proper dissolution, transferred to an amber vial, and stored at room temperature, where it was stored for up to 6 months. A taurine solution (0.1 M) was prepared in phosphate/borate buffer (0.01 M; pH 8.0) and stored for up to 3 months at room temperature. An Ellman’s reagent solution (0.01 M) was prepared in ethanol, pyrrolidine solution (0.01 M) was prepared in deionized water, and an MBB solution (0.04 M) was prepared phosphate/borate buffer (in 0.01 M; pH 8.0). These solutions were stored at 4 °C in the dark up to 3 months, 2 weeks, and 4 days, respectively. NH_4_OH (5%) was prepared from NH_4_OH (29%) and diluted with MeOH. ATCA-^13^C, ^15^N_2_ was prepared and diluted in methanol to produce a 50 µM standard. Ammonium acetate (0.005 and 0.03 M) was prepared in deionized water. These solutions were stored at 4 °C. KCN and KSCN stock solutions (10 mM each) were prepared in 10 mM NaOH and diluted into a 1 mM mixture of KCN and KSCN with 10 mM of aqueous NaOH. The obtained mixture was diluted to the desired working concentrations with 10 mM aqueous NaOH for all standards. The calibration standards for CN and SCN^−^ were prepared at concentrations of 10–500 µM and 5–200 µM, respectively. The calibration standards for ATCA were prepared and at concentrations of 30–300 µM. Prior to use, the working standard solutions for CN, SCN^−^, and ATCA were prepared by diluting the suitable stock solutions with swine postmortem blood to achieve the desired concentrations.

Caution: It is crucial to be aware that CN poses a significant risk to both humans and animals due to its highly toxic nature. Therefore, handling CN solids and solutions requires utmost care and caution. When solutions fall below a pH of about 10 (i.e., pKa of CN is 9.2), CN is released as HCN(g). To prevent this potentially hazardous situation, all aqueous standards containing CN were prepared in a 10 mM NaOH solution, ensuring the non-volatile state of CN. Furthermore, all CN solutions were exclusively handled within a well-ventilated hood.

### Methods

#### CN exposures

Note: The animals used for this study were control animals used in a separate research study for the evaluation of a potential next-generation cyanide antidote. Our study utilized blood samples from these animals to add value to the drug development study under ethical principles of maximizing the utility of existing samples and minimizing the need for additional animal use.

Blood samples from eight pigs (Species: *Sus scrofa*; Breed: Yorkshire cross; Weight: 45–55 kg) were obtained at the Department of Emergency Medicine at the University of Colorado (Anschutz Medical Campus, Aurora, CO). Before CN exposure, blood samples were drawn to establish a baseline (“zero”) time point. Pigs were then exposed to intravenous CN at 0.2 mg/kg/min and after meeting euthanization criteria, were euthanized with sodium pentobarbital. Two pigs (Pigs 2 and 8) met euthanasia criteria earlier, at 40 and 38 min following CN exposure, respectively. Previous cyanide toxicity studies demonstrated that a sustained mean arterial blood pressure of less than 30 mmHg for 10 min is terminal. Animals meeting this criteria were euthanized. All other animals were euthanized after a 90-min observation period. Heparin (10,000 units) was administered in an attempt to limit coagulation of the animal’s blood postmortem. Following euthanasia, animals were transferred to a walk-in cold room where they were stored at 4 °C to obtain blood samples. Blood samples were collected from a central venous catheter placed in the external jugular as follows: the catheter was flushed with 5 mL normal saline to clear the catheter, 10 mL of blood was collected and discarded to minimize the potential for contamination of the sample, then a 3-mL blood sample was collected for analysis. Following blood collection, the jugular catheter was flushed with 10 mL of normal saline followed by 3 mL of heparin to prevent clotting within the catheter. Blood samples were collected into heparinized blood collection tubes at 1, 2, 4, 24, 48, 72, 120, and 168 h after death, where possible. Despite heparinization, clotting blood blocked most catheters before the end of the experiment. Table 1 shows the blood samples collected for each pig and the *N* value at each time point. Following collection, blood samples were flash-frozen in liquid nitrogen, stored in a –80 °C freezer, and then shipped on dry ice to South Dakota State University. Upon receipt, all blood samples were stored at −80 °C until analyzed.

**Table 1:** Sampling time points following euthanasia where postmortem blood was collected for the analysis of CN, SCN^−^, and ATCA in a group of eight pigs

Pigs (45–55 kg)	“Background” Blood draw	Blood draw time following euthanasia (h)							
		1	2	4	24	48	72	120	168
1	✓	✓	✓	✓	✓				
2	✓	✓	✓	✓					
3	✓	✓	✓	✓	✓	✓	✓	✓	✓
4	✓	✓	✓	✓					
5	✓	✓	✓	✓	✓	✓	✓		
6	✓	✓	✓	✓					
7	✓	✓	✓	✓	✓	✓	✓		
8	✓	✓	✓	✓					
*N* value	8	8	8	8	4	3	3	1	1

“Background” indicates a blood drawn from the pig prior to CN exposure for the purpose of analyzing the endogenous levels of CN, SCN^−^, and ATCA. A check mark (✓) indicates the successful collection of a blood sample at that specific time point for the corresponding pig

All animals were cared for in compliance with the “Principles of Laboratory Animal Care” formulated by the National Society for Medical Research and the “Guide for the Care and Use of Laboratory Animals” prepared by the National Institutes of Health (National Academic Press, 1996). The University of Colorado's Institutional Animal Care and Use Committee (IACUC) approved the CN exposure study which complied with the regulations and guidelines of the Animal Welfare Act and the American Association for Accreditation of Laboratory Animal Care.

#### Preparation of CN and SCN^−^ for analysis

Postmortem blood samples were prepared for HPLC–MS/MS analysis of CN and SCN^−^ following the established method developed by Alluhayb et al. [50] where detailed instructions and additional information can be found. Briefly describing the method, the blood samples were thawed at room temperature, vortexed for 1 min, and centrifuged at 3600×*g* (Sorvall™ Legend™ X1 Centrifuge with TX-400 Rotor Adapter, Thermo Scientific™) prior to analysis. Postmortem blood (100 μL of control or sample) was obtained and subsequently spiked with a Na^13^C^15^N and NaS^13^C^15^N mixed internal standard solution (500 μM each, 10 μL). After thorough vortexing, the blood sample was divided into two portions for CN (25 μL) and SCN^−^ (75 μL) sample preparation.

Cyanide was prepared for analysis using active microdiffusion. Reagent solutions (200 µL each) containing NDA (2 mM), taurine (100 mM), and pyrrolidine (10 mM) were added to the reagent chamber of a two-chamber sample preparation cartridge. Postmortem blood (25 µL) was added to the sample chamber and diluted with 50 µL of water. Aqueous H_2_SO_4_ (200 µL) was added to the sample chamber. The sample and reagent chambers were sealed, and air was passed through the sample chamber into the capture chamber. This facilitated the transfer of HCN(g) to the capture solution. Within the capture chamber, the CN reacted with NDA and taurine to form a CN-NDA-taurine complex. For SCN^−^, postmortem blood (75 µL) was treated with ACN (1000 μL) to precipitate proteins. After vortexing for 1 min and centrifugation for 10 min (−5 °C) at 16,700x*g* (Sorvall™ Legend™ Micro 21R Microcentrifuge with 24 × 1.5/2.0 mL Rotor with ClickSeal™ Biocontainment Lid, Thermo Scientific™), the supernatant (750 μL) was transferred and dried. The dried sample was reconstituted with 10 mM aqueous ammonium formate (75 μL). Ellman’s reagent (10 mM, 50 μL) was added to react with free thiols, followed by vortex mixing. Subsequently, MBB (4 mM, 50 μL) was added to form the SCN-bimane complex.

The prepared CN and SCN^−^ solutions, 550 μL of the CN capture chamber solution and 175 μL of the prepared SCN^−^ solution, were combined for subsequent HPLC–MS/MS analysis in a 4-mL glass screw top vial. The mixture was then filtered through a 0.22-μm polytetrafluoroethylene (PTFE) filter into a 150-μL glass insert for follow-on HPLC–MS/MS analysis.

Note that multiple species of cyanide exist. CN is used in this manuscript to denote any and all cyanide species (i.e., including CN^−^(aq), HCN(aq), and HCN(g)). This is done because cyanide speciation is complex where CN^−^ exists in blood and urine, but with the pH of blood (approximately 7.2) and the pKa of HCN(aq) (approximately 9.2), HCN(aq) is the dominant form of “free” cyanide [i.e., in a simple aqueous system, over 99% of the cyanide exists as HCN(aq)]. While HCN(aq) is the predominant species in solution, cyanide can also be protein bound (i.e., it reacts with cysteine residues), it can be bound to hemoglobin and can react with glutathione, and there may be microenvironments where the pH is higher and cyanide may exist as CN-. Since the blood is acidified during the sample preparation, the method here detects a combination of all the cyanide species.

### Preparation of ATCA for analysis

Postmortem blood samples were prepared for HPLC–MS/MS analysis of ATCA following the established method of Giebułtowicz et al. [49] with slight modifications. Briefly, the blood samples were thawed, vortexed, and centrifuged at 3600×*g* prior to analysis. Postmortem blood (100 µL of control or sample) was placed in an Eppendorf test tube and mixed with DI water (100 µL) for 1 min by hand, followed by 3 min of vortexing. Cold ACN (750 µL) was added, vortexed for 2 min, and placed in a freezer for 20 min at –20 °C. Then the sample was centrifuged for 5 min at 16,700xg. Subsequently, the supernatant (800 µL), H_3_PO_4_ (85%, 20 µL), ATCA-^13^C, ^15^N_2_ (100 µM, 50 µL), and ACN (130 µL) were combined in a separate Eppendorf test tube containing solid MCX sorbent (10 mg). The mixture was then placed on a laboratory shaker for 5 min. The sorbent was washed separately with ACN (1 mL) and ammonium acetate (500 µL of 0.03 M), with vortexing for 1 min and centrifugation for 5 min at 16,700x*g* after each wash. Subsequently, NH_4_OH (5%, 500 µL) was added and vortexed for 30 min to elute the ATCA from the MCX sorbent. The supernatant (450 µL) was transferred to a glass test tube and evaporated to dryness using a nitrogen stream at 40 °C. The sample was then reconstituted with ACN (350 µL) and ammonium acetate (0.005M, 15 µL) and transferred to an HPLC–MS/MS vial for analysis.

#### HPLC–MS/MS conditions for analysis of CN, SCN^−^, and ATCA

Prepared samples were analyzed using a Shimadzu HPLC (LC20AD, Shimadzu Corp., Kyotu, Japan) coupled with an AB Sciex Q-trap 5500 triple quadrupole mass spectrometer (Applied Biosystems, Foster City, CA, USA) to detect CBI (N-substituted 1-cyano [f] benzoisoindole) and SCN-bimane using negative ionization, and ATCA using positive ionization. Data acquisition and peak integration for analytes were carried out using Analyst™ software 1.4.1. (Framingham, MA, USA).

For CBI and SCN-bimane, HPLC was conducted with a ZORBAX RRHT Eclipse Plus C_18_ column (100 mm × 3.0 mm, 1.8 μm, 95 Å) protected by a ZORBAX RRHD Eclipse Plus C_18_ UHPLC guard column (5 mm × 3.0 mm, 1.8 μm, 95 Å) (both from Agilent Technologies, California, USA). For the separation of CBI and SCN-bimane, a gradient of 10 mM ammonium formate in water (Mobile phase A) and 10 mM ammonium formate in methanol (Mobile phase B) was used. The details of the method, including MS parameters, are given in Alluhayb et al. [50].

For ATCA, an Atlantis Premier BEH Z-HILIC Column (50 mm × 2.1 mm, 5 µm) (Waters Corporation, Milford, MA) was used. The mobile phases consisted of HPLC-grade water with 0.1% formic acid as eluent A and acetonitrile with 0.1% formic acid as eluent B at a flow rate of 0.5 mL/min and 40 °C. Detailed MS parameters are given in Giebułtowicz et al. [49].

#### Stability kinetics

Kinetic parameters were assessed using the methodologies established and detailed by the World Health Organization and Shargel et al. [51, 52]. The concentration–time curves were utilized to calculate the elimination half-life (*t*_1/2_) and the elimination constants (*K*_e_) through extrapolation. To determine the ratio *C*_max_/*C*_baseline_, the maximum blood concentration was divided by the endogenous (baseline) concentration.

#### Stability of CN, SCN^−^, and ATCA in sampled postmortem blood

To evaluate the stability of CN, SCN^−^, and ATCA in postmortem blood under potentially important conditions for forensic analysis, we stored aliquots of the 2-h postmortem blood sample at room temperature (RT) and typical human body temperature (HBT) and measured CN, SCN^−^, and ATCA immediately following, and 1, 2, 4, 8, 12, 24, 48, 72, 120, 168, and 240 h (10 days) of storage. The stability of the biomarkers of CN was calculated as a percentage of the initial concentration of the respective analyte.

## Results

Pigs in the study were exposed to a potentially lethal concentration of CN and were euthanized either when they met approved criteria or after a 90-min observation period. Following this CN exposure, the initial postmortem blood sample, obtained 1 h postmortem, showed dramatic escalations of blood concentrations of CN and its metabolites compared to background in postmortem swine samples. Specifically, CN's half-life (*t*_1/2_) was observed to be 34.3 h, indicating a rapid decline in concentration postmortem. In contrast, SCN^−^ (*t*_1/2_ = 359 h, 15 days) and ATCA (*t*_1/2_ = 544 h, 23 days) demonstrated significantly greater stability.

The pattern of instability in CN was accentuated under varying storage temperatures. At room temperature (RT, 25 °C), CN’s half-life (*t*_1/2_) was reduced to 10.7 h, and at human body temperature (HBT, 37 °C), it declined further to 6.6 h. This resulted in CN concentrations falling below baseline levels within 50, 74, and 120 h for postmortem swine stored at 4 °C, and in postmortem blood at RT and HBT, respectively. Conversely, SCN^−^ and ATCA exhibited far greater stability under these conditions. For postmortem swine, SCN^−^’s half-life (*t*_1/2_) was 15 days, and ATCA’s was 23 days. For blood stored at RT and HBT, SCN^−^’s half-lives (*t*_1/2_s) were 18–19 days, and ATCA’s were 24–27 days.

**Fig. 2:**
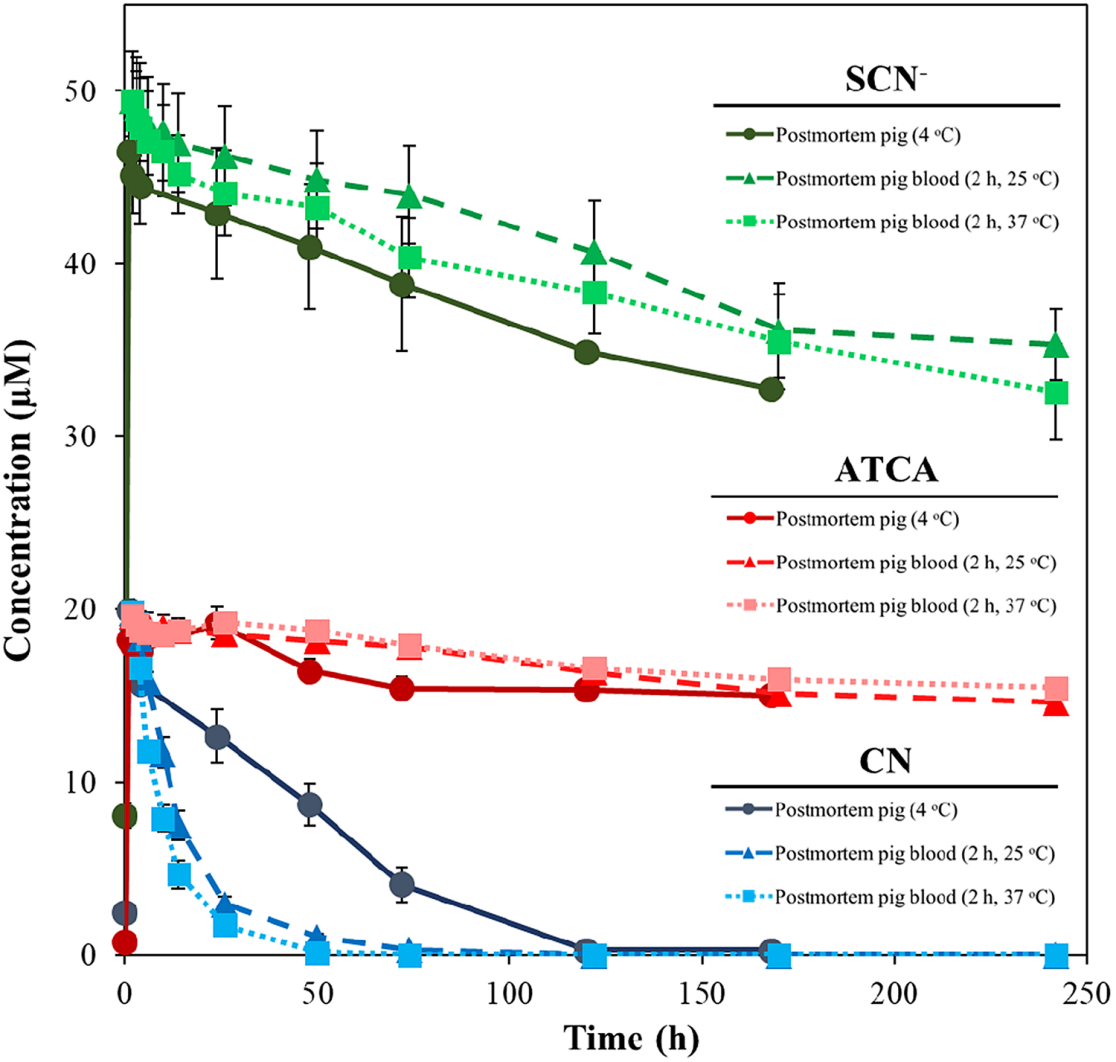
The stability of CN, SCN^−^, and ATCA in postmortem CN-exposed swine. The circles connected via solid lines represent the stability of these compounds in postmortem pigs. Blood stored at room temperature (25 °C) is indicated by triangles and dash lines, while blood stored at a typical body temperature (37 °C) is shown by squares connected by dotted lines. Error bars represent standard error of mean (±SEM) (*N* = 8)

Figure 2 in the study presents the stability of CN, SCN^−^, and ATCA in postmortem CN-exposed swine under these different conditions, differentiating storage at RT and HBT. Error bars in the figure represent the standard error of mean (± SEM) for each set of measurements (*N* = 8). Table 2 provides detailed stability and kinetic parameters for CN, SCN^−^, and ATCA in postmortem blood of CN-exposed pigs stored at 4 °C and in postmortem swine blood stored at RT and HBT. It includes environment-specific half-lives and the ratios of the initial concentration 1 h postmortem (*C*_1h_) to the background concentrations (*C*_background_) for each biomarker.

**Table 2:** Stability/kinetic parameters for CN, SCN^−^, and ATCA in postmortem blood of CN-exposed pigs (stored at 4 °C) and in postmortem swine blood stored at 25 °C and 37 °C

Environment	Analyte	*t*_1/2_ (h)	*t*_1/2_ (day)	*C*_1h_/*C*_background_
Pig (stored at 4 °C)	CN	34.3	1.4 d	11
	SCN^−^	359	15 d	5.2
	ATCA	544	23 d	26
2 h postmortem pig blood (stored at 25 °C)	CN	10.7	0.5 d	NA
	SCN^−^	432	18 d	
	ATCA	573	24 d	
2 h postmortem pig blood (stored at 37 °C)	CN	6.63	0.3 d	NA
	SCN^−^	450	19 d	
	ATCA	653	27 d	

## Discussion

### Stability of CN, SCN^−^, and ATCA in postmortem CN-exposed pigs

Following CN exposure, the initial postmortem blood sample, obtained 1 h postmortem, showed dramatic escalations of blood concentrations of CN and its metabolites compared to background. Table 2 lists the ratios of the initial concentration of each CN biomarker (i.e., 1 h postmortem blood) compared to its respective background concentration (*C*_1h_:*C*_background_) (i.e., concentrations from antemortem blood collected from the same animal prior to CN exposure). The *C*_1h_:*C*_background_ ratio decreases from ATCA > CN > SCN^−^. Therefore, ATCA is the most elevated marker of CN exposure compared to its background concentration even though SCN^−^ has the highest absolute blood concentration at this time. A higher *C*_1h_:*C*_background_ ratio is advantageous for use as a marker of CN exposure since it is easier to differentiate elevated levels from background concentrations. The *C*_1h_:*C*_background_ ratio for SCN^−^ is approximately five times lower than for ATCA because of the relatively high background concentrations of SCN^−^. The high background concentrations of SCN^−^ were expected based mainly on dietary exposure to SCN^−^. While the increase for all markers of CN exposure at 1 h could be interpreted as evidence that each of these markers could be used to confirm acute exposure to lethal CN concentrations, it is important to note that the conditions in this study were ideal for preserving forensic information. These conditions include animals housed under controlled conditions (including a controlled diet), known background concentrations of each marker for each animal, immediate storage of animals at 4 °C following euthanasia, and deliberate handling of blood samples with immediate freezing and subsequent storage at −80 °C until analysis. In a more realistic situation, a victim’s diet is not controlled, the background concentrations of these biomarkers are not available, and it is unlikely that blood samples are immediately gathered and properly stored. It should be noted that CN drops below its background concentrations at 120 h. This is likely because generation of CN from diet or normal metabolism has ceased, and redistribution occurs postmortem.

Following the immediate sharp rise in the concentration of CN, it relatively rapidly decreases to baseline levels by 120 h, whereas ATCA and SCN^−^ exhibit a steady, slow, decrease in the postmortem swine over the course of the experiment, maintaining well above the initial blood concentration. As shown in Figs. 2, S1A, and S2A, CN decreased markedly faster than SCN^−^ and ATCA, producing the lowest *t*_1/2_  =  34.3 h of the markers of CN exposure (Table 2 and Figure S2A). The rapid decrease in CN concentration is consistent with multiple other studies which also observe a rapid decrease in CN concentrations in blood under a variety of storage conditions [53–58]. The inherent instability of CN is problematic for its use as a forensic biomarker of CN poisoning.

The alternative markers of CN exposure analyzed in this study, SCN^−^ and ATCA, showed more stability versus CN in postmortem pigs, but the half-life of ATCA (*t*_1/2_ = 544.3, *h* = 23 d) was nearly double that of SCN^−^ (*t*_1/2_ = 326.4, *h* = 15 d) (Table 2 and Fig. S2A). The increased stability of SCN^−^ and ATCA is advantageous for forensic purposes, allowing for a longer window-of-opportunity to verify if the cause of death is CN poisoning. Therefore, because ATCA is the most stable marker of CN poisoning, it is more advantageous for forensic purposes, especially if ideal conditions cannot be achieved.

### Stability of CN, SCN^−^, and ATCA in postmortem blood at elevated temperatures

Under more typical circumstances of CN poisoning, deceased victims would not be immediately transitioned to cold storage (4 °C). In addition, the time between a poisoning event and cooled storage may be lengthy and/or cooled storage may not even occur. Therefore, the postmortem stability of CN, SCN^−^, and ATCA in victims of CN poisoning at RT and HBT is of interest. Figure 2 shows the stability of CN, SCN^−^, and ATCA in 2 h postmortem blood samples of CN-exposed pigs stored at RT (dashed lines) and HBT (dotted lines) over 10 days (240 h). CN exhibited pronounced instability at these temperatures, measured as a rapid decrease in CN concentrations with near complete elimination by 74 h, as illustrated in Fig. 2, S1B, S1C, and S2C. The stability of CN in postmortem blood at RT and HBT is much lower than in postmortem swine stored at 4 °C. The *t*_1/2_s of CN at RT and HBT (Table 2 and Figure S2B and C) show the rapid degradation of CN in postmortem blood at elevated temperatures. Compared to postmortem swine at 4 °C, postmortem blood produces a 3× and 5× decrease in *t*_1/2_ for RT and HBT, respectively.

The stability of SCN^−^ and ATCA in postmortem blood (at RT and HBT) was much higher than CN (Figs. 2, S1B, and S1C). To quantify this relationship, the *t*_1/2_s for each biomarker and storage condition pair are reported in Table 2. The *t*_1/2_s for SCN^−^ and ATCA are approximately 40× and 53× higher than CN at RT, respectively, and 68× and 99× higher than CN at HBT for SCN^−^ and ATCA, respectively. In a real-world scenario, where ideal conditions are rare, this study indicates that ATCA and SCN^−^ offer more persistent markers of CN poisoning, with ATCA being about 1.5× more stable than SCN^−^.

### ATCA is a reliable postmortem biomarker of CN poisoning

The relatively low stability of CN in postmortem CN-exposed pigs and in postmortem blood at RT and HBT presents a major problem for direct analysis of CN as a marker of CN poisoning. Specifically, CN concentrations rapidly declined, particularly at RT and HBT. The rapid degradation of CN can lead to challenges in forensic analyses, particularly when trying to ascertain whether an individual was exposed to lethal concentrations of CN. The inherent instability of CN not only affects its potential as a consistent marker but also casts a shadow on the reliability of forensic analysis of CN when CN poisoning is suspected as a cause of death. Thus, if direct analysis of CN is to be used for this purpose, immediate sample collection, proper storage, and timely analysis are imperative to ensure trustworthy forensic information.

The degradation behavior of SCN^−^ and ATCA was comparable under the conditions tested, but ATCA was about 1.5× more stable. Although SCN^−^ can be considered to have acceptable stability, the major disadvantage of SCN^−^ for forensic analysis is its high and variable endogenous concentrations (i.e., SCN^−^ produced the highest background concentrations but the most variable). Elevated concentrations of SCN^−^ relative to CN and ATCA are based on efficient metabolism of CN and SCN^−^ consumed via diet. The dependence of background concentrations of SCN^−^ on diet is disadvantageous since it is more difficult to determine if elevated SCN^−^ concentrations are produced from CN exposure. Further, while the absolute concentrations of SCN^−^ following CN exposure were the largest in this study (Fig. 2), the increase in SCN^−^ relative to its baseline concentration was small compared to CN and ATCA (Table 2). Factors such as age, health, genetics, and gut microbiota also influence the conversion of CN to SCN^−^, leading to increased variability among individuals. Therefore, SCN^−^ suffers from large and variable background concentrations and limited increase relative to its respective background concentration. Therefore, while SCN^−^ can be a valuable marker for assessing CN exposure in the context of other evidence, elevated concentrations are likely not conclusive evidence of CN poisoning under most conditions.

2-Aminothiazoline-4-carboxylic acid demonstrated the slowest elimination dynamics among the three analytes, maintaining concentrations nearest the initial postmortem swine blood draw (1 h) throughout the study (10 days) while it was the most stable marker, it still decreased over time under all study conditions. This decrease is likely be due to redistribution of ATCA following death or changes in the postmortem blood matrix. Regardless of the time elapsed and inherent postmortem blood transformation over time, ATCA levels remain remarkably consistent. This distinguishes it from CN and SCN^−^, which can show more significant degradation. Moreover, the large increase in ATCA above background levels is advantageous for forensic purposes, providing a longer runway to confidently determine elevated levels of ATCA. Based on its properties of detectability, stability, and relative increase directly related to CN exposure, ATCA can be used as a forensic marker to verify CN exposure.

While this study answered an important question in the field of forensic analysis related to CN poisoning, there are other questions important to definitely answer: (1) “Is ATCA the most advantageous marker when blood is obtained from the heart (i.e., the heart is a common location to draw blood postmortem)?,” (2) “Is ATCA the most appropriate marker when death from CN poising is delayed (i.e., when active metabolism of ATCA occurs over a significant amount of time)?,” and (3) “What effect would tissue decay have on the postmortem concentrations of ATCA?.” There have been studies that shed light on question 2, such as Bhandari et al., [3] which showed ATCA tracked CN concentrations, producing a *t*_1/2_ of only 40.7 and 13.9 min in rabbits and swine, respectively. ATCA concentrations decline faster when metabolism is active because it is excreted through normal metabolic pathways. Overall, active metabolism directly impacts the persistence of ATCA. Therefore, confirmation of ATCA’s behavior during active metabolism must be considered. For question 3, our study did not address the effect of biological decay on the behavior of CN, SCN^−^, or ATCA. For example, after death, CN can continue to be produced due to various biological processes [41, 55, 59–63]. This postmortem production of CN could lead to formation of ATCA in the presence of cysteine. If this process is common, ATCA measured in postmortem blood may not necessarily originate from CN poisoning. Future research is needed to address these questions to solidify the situations where the use of ATCA as a forensic biomarker for cyanide exposure in forensic investigations is appropriate.

## Conclusion and future work

The findings of this study highlight the advantages of ATCA as a biomarker of CN poisoning based on its relative stability in postmortem pigs at 4 °C and in postmortem blood at RT and HBT. ATCA demonstrated the slowest elimination and produced a high *C*_1h_:*C*_background_ ratio. The slow and predictable decline of ATCA over extended intervals provides a long window-of-opportunity to detect ATCA above background levels during postmortem analysis of blood from suspected CN poisoning victims, particularly in real-world scenarios, where ideal conditions are rare. Given its pronounced postmortem stability, ATCA emerged as the best candidate for use as a biomarker of CN poisoning.

### Supplementary Information

Below is the link to the electronic supplementary material.

Supplementary file1 (DOCX 374 kb)
