# Supplementary material for: Can the cyanide metabolite, 2-aminothiazoline-4-carboxylic acid, be used for forensic verification of cyanide poisoning?
Source: Forensic Toxicol. 2024 May 13;42(2):221–31. doi: 10.1007/s11419-024-00690-4 (PMC11269370; doi:10.1007/s11419-024-00690-4)
Supplement: Supplementary file 1 — Supplementary file1 (DOCX 374 kb) [file 11419_2024_690_MOESM1_ESM.docx]

**SUPPORTING INFORMATION**

**Can Cyanide Metabolite 2-Aminothiazoline-4-Carboxylic Acid Be Used to Verify Cyanide Poisoning in Postmortem Blood?**

**Authors:** Abdullah H. Alluhayb^a^, Carter Severance^b^, Tara Hendry-Hofer^b^, Vikhyat S. Bebarta^b^, and Brian A. Logue^a^*

^a^Department of Chemistry and Biochemistry, South Dakota State University, Box 2202, Avera Health and Science Center 131, Brookings, SD, 57007, USA

^b^Department of Emergency Medicine, University of Colorado School of Medicine, Aurora, CO 80045, USA

***Corresponding Author:** Brian.Logue@sdstate.edu

Supplementary Figure S1A demonstrates the superior stability of ATCA compared to CN and SCN⁻ in postmortem swine (stored at 4 °C) following CN poisoning. While CN concentrations rapidly decreased to near zero by 120 h, and SCN⁻ declined to 70% and ATCA reduced to 82% of their initial concentrations by 168 h. These data highlight the relative stability of CN, SCN⁻, and ATCA in postmortem swine over extended storage times. The marked stability of ATCA supports its use as a reliable biomarker for CN poisoning confirmation.


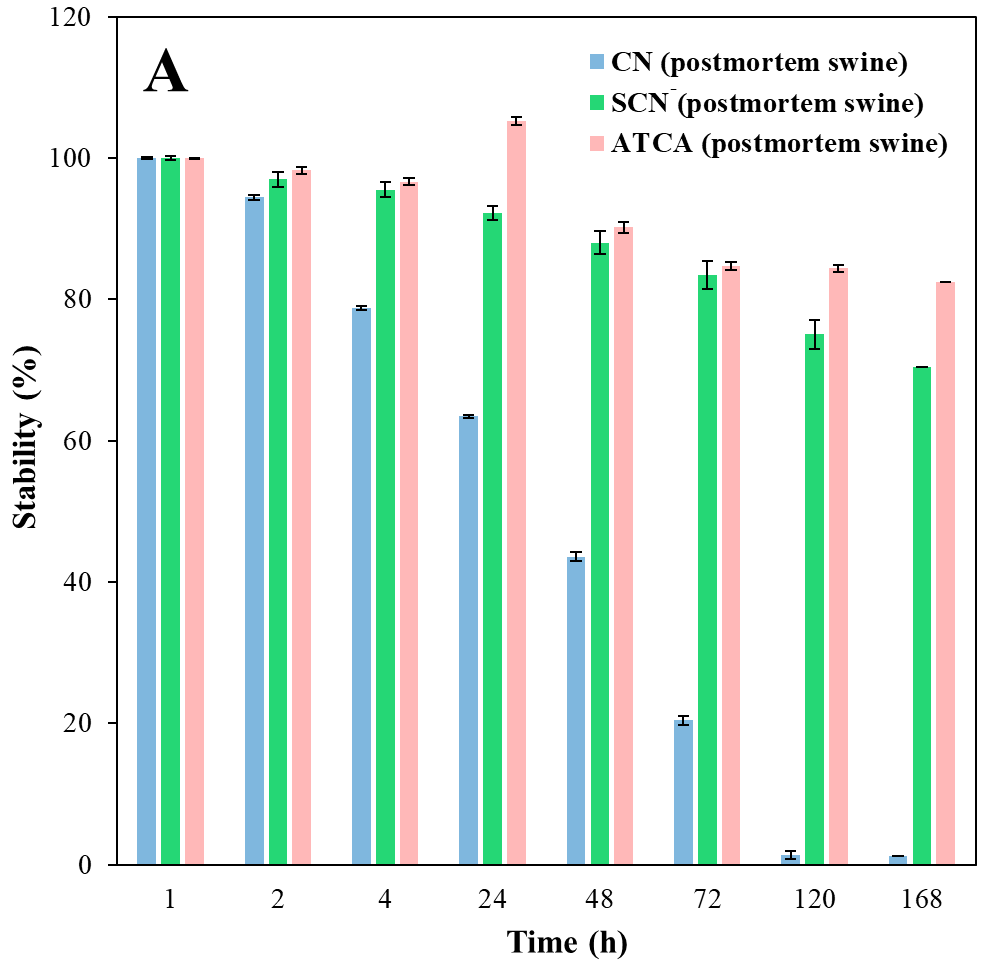


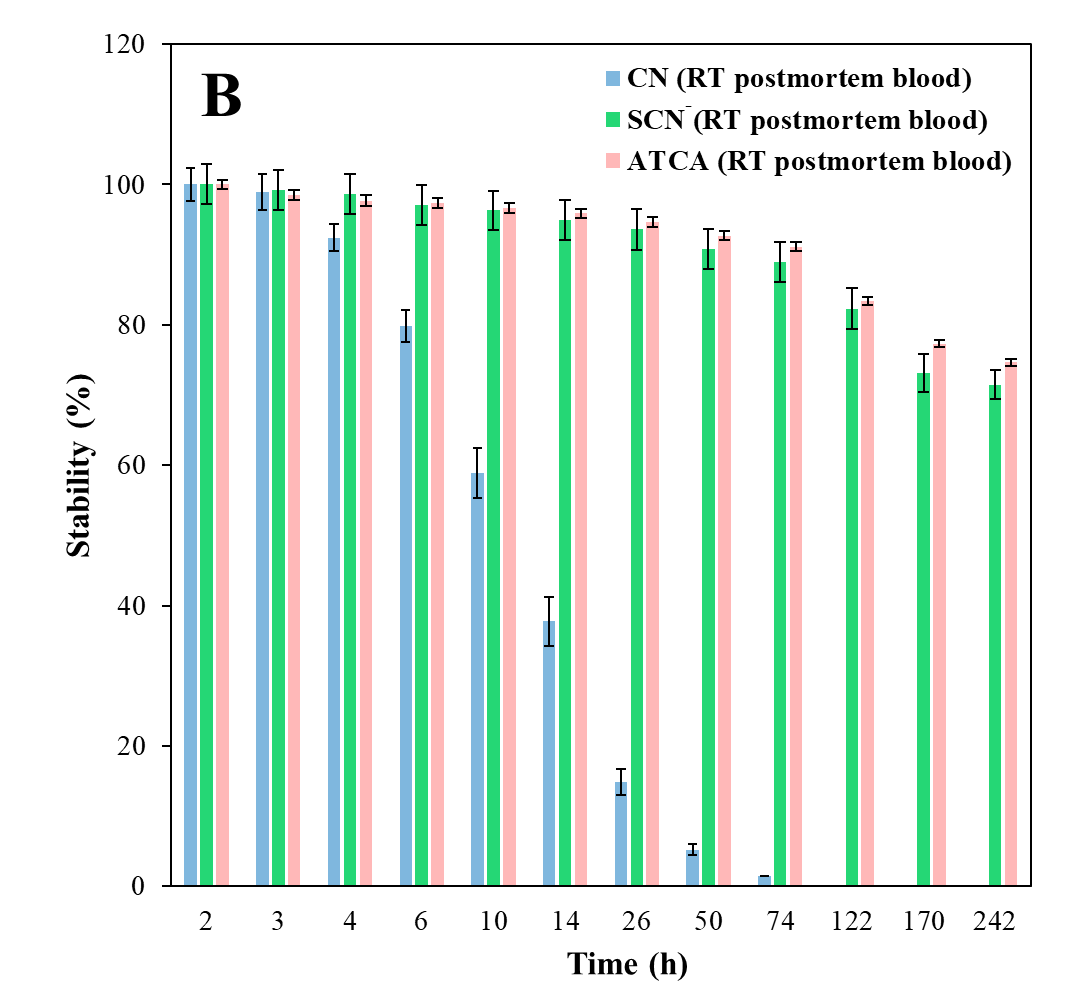


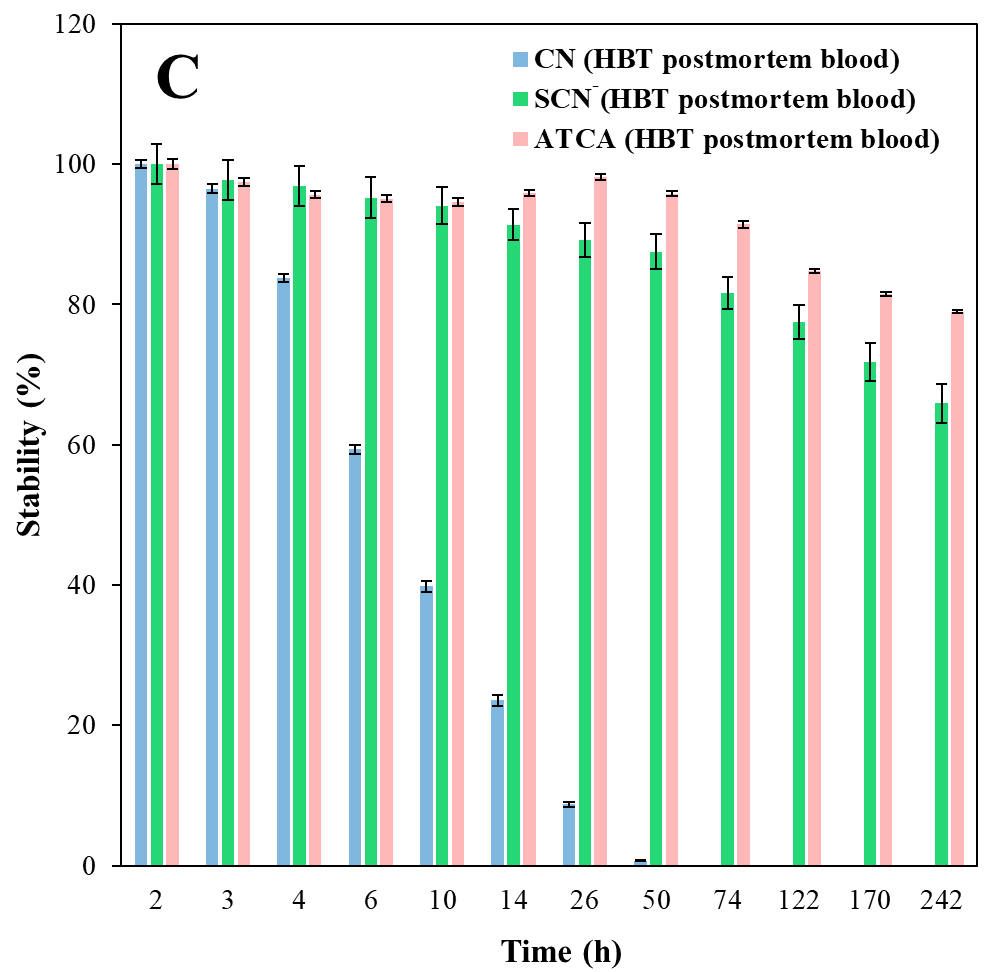


**Figure S1.** The stability of CN, SCN⁻, and ATCA in A) postmortem CN-exposed swine; B) room temperature; C) and typical body temperature. Error bars are plotted as standard error of mean (± SEM) (N = 8)

Supplementary Figures S1B and S1C show the stability of CN, SCN⁻, and ATCA over time in postmortem blood stored at RT and HBT, respectively. The rapid degradation of CN is clearly illustrated, with concentrations decreasing to near zero by 74 h and 50 h at RT and HBT, respectively. SCN⁻ and ATCA decline more slowly, however the stability of ATCA is markedly better than SCN⁻ at HBT. At RT, ATCA and SCN⁻ maintain concentrations at 74 h. After 74 h (at HBT), ATCA declined to 79% of its initial concentration but SCN⁻ dropped to 66%. Together these figures demonstrate the stability of SCN⁻ and ATCA under elevated storage conditions compared to the inherent instability of CN.

Figure S2A shows the calculation of *t_1/2_* for CN, SCN⁻, and ATCA in postmortem swine blood stored at 4°C. It is clear that CN has the shortest *t_1/2_* of 34.3 h. Though SCN⁻ is more stable than CN, ATCA exhibits the longest the *t_1/2_* of 23 days nearly double that of SCN⁻ 15 days. These *t_1/2_* values highlight the instability of CN versus the persistence of ATCA as a forensic biomarker.


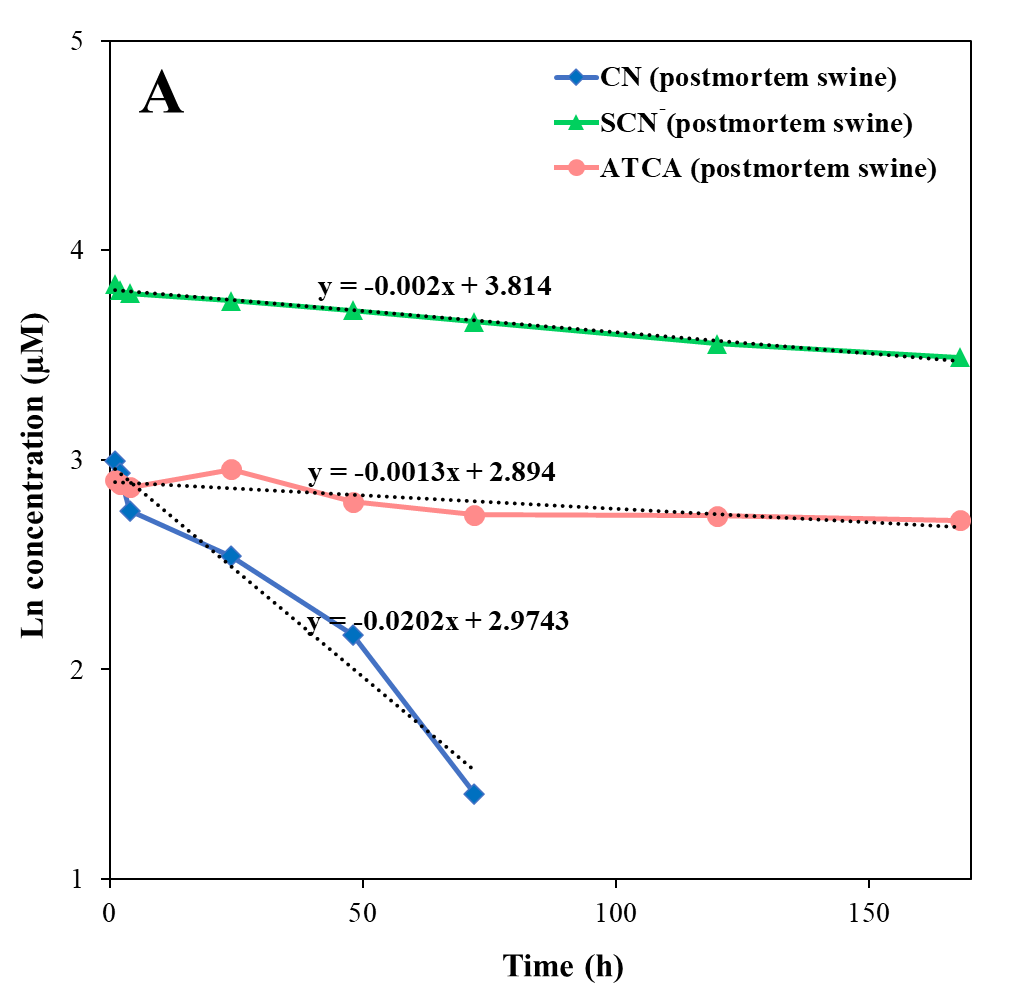


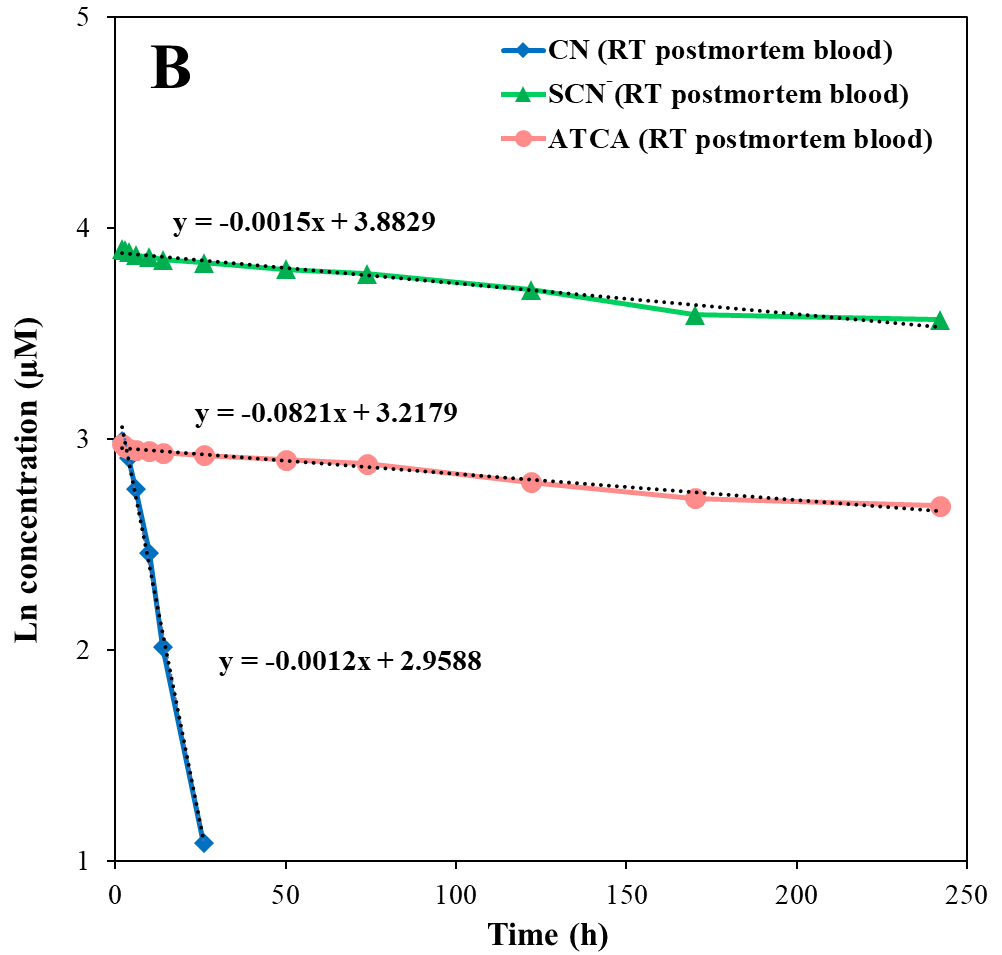


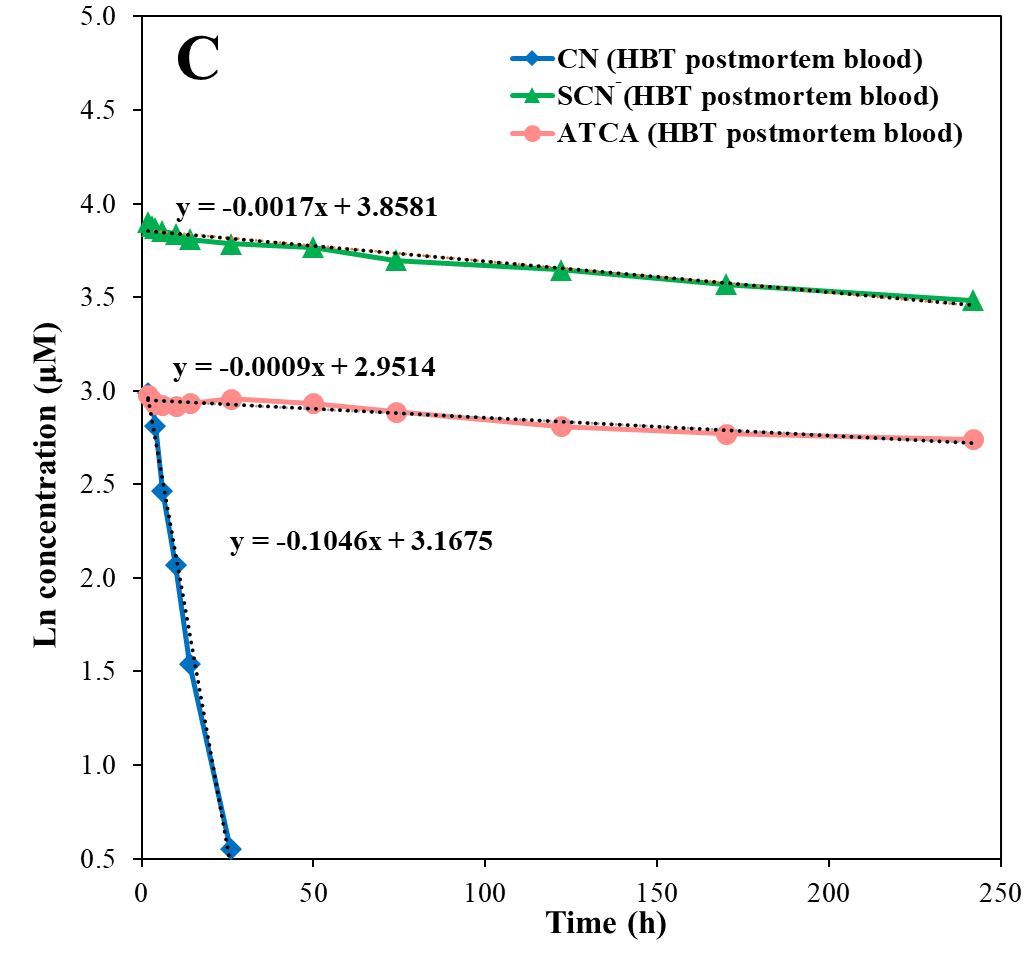


**Figure S2.** The t_1/2_s for CN, SCN⁻, and ATCA in A) postmortem CN-exposed swine; B) room temperature; C) and typical body temperature.

The rapid degradation of CN is further demonstrated in Supplementary Fig S1B and C, which show the *t_1/2_*s calculation for CN stored in postmortem blood at RT and HBT. As shown in Table 2 and Figures S2B and C, the *t_1/2_* of CN drops to 10.7 h and 6.6 h at RT and HBT, respectively. Meanwhile, SCN⁻ and ATCA remain much more stable, with *t_1/2_* values of 18 and 24 days (RT) and 19 and 27 days (HBT) (i.e., SCN⁻ is ≈40-68x and ATCA is ≈53-99x more stable than CN for these temperature conditions). This quantitative comparison of *t_1/2_* values under different storage conditions highlights the advantages of ATCA as the most stable forensic marker of CN poisoning in postmortem swine.
